# Revisiting alpha-theta cross-frequency dynamics during working memory

**DOI:** 10.1093/cercor/bhaf344

**Published:** 2026-01-13

**Authors:** Julio Rodriguez-Larios, Mark J Roberts, Saskia Haegens

**Affiliations:** Department of Psychology, Brunel University London, Kingston Lane, Uxbridge UB8 3PH, England, United Kingdom; Department of Cognitive Neuroscience, Faculty of Psychology and Neuroscience, Maastricht University, Oxfordlaan 55, 6229 EV Maastricht, The Netherlands; Department of Psychiatry, Columbia University Irving Medical Center, Herbert Pardes Building, 1051 Riverside Drive, New York, NY 10032, United States; Division of Systems Neuroscience, New York State Psychiatric Institute, 1051 Riverside Drive, New York, NY 10032, United States

**Keywords:** connectivity, cross-frequency coupling, eeg, neural oscillations, working memory

## Abstract

Prior EEG research has shown that during working memory, alpha (8 to 14 Hz) and theta (4 to 8 Hz) oscillations tend to form a 2:1 frequency ratio. According to the Binary Hierarchy Brain Body Oscillation Theory (BHBBOT), a recent model grounded in mathematical analysis, this cross-frequency configuration reflects enhanced connectivity between brain regions generating these rhythms. However, this prediction has not yet been empirically tested. In this study, we leveraged high density EEG, source localization and connectivity metrics derived from Information Theory (IT) and the Theory of Weakly Coupled Oscillators (TWCO) to examine whether the previously observed alpha-theta cross-frequency dynamics during working memory are accompanied by changes in connectivity. Our results show that a significant increase in the proportion of 2:1 ratios between regions generating frontal theta and parietal alpha rhythms was accompanied by relative decreases in connectivity, as revealed by both IT and TWCO metrics. Furthermore, phase synchrony between these two regions was significantly reduced during working memory and correlated negatively with behavioral performance. In conclusion, our results show that the increased occurrence of 2:1 alpha:theta cross-frequency ratios during working memory reflects functional segregation (rather than integration) and therefore directly challenges some of the predictions of the BHBBOT.

## Introduction

Cognitive functions require the coordinated activity of many brain areas (Christophel et al. 2017), and neural oscillations are thought to support this (X.-J. Wang 2010). Brain oscillations are rhythmic patterns of neural activity that create sequences of excitation and inhibition (Buzsáki and Draguhn 2004). It has been proposed that neural oscillations gate inter-areal communication by the alignment of excitatory phases (or “duty cycles”) that make both spike output and sensitivity to synaptic input coincide in time (Varela et al. 2001; Fries 2015). In support of this idea, phase locking between areas oscillating at a similar frequency has been shown to be behaviourally relevant in both human and animal neurophysiology (Spyropoulos et al. 2018; Vissani et al. 2025).

Different brain areas generate oscillations at different frequencies (Capilla et al. 2021) depending on the neurophysiological characteristics of their neural populations (von Stein and Sarnthein 2000; Buzsáki and Draguhn 2004; Lea-Carnall et al. 2016). The interaction between areas that oscillate at different frequencies can be captured by various cross-frequency coupling metrics (eg phase-phase, phase-amplitude, amplitude-amplitude) (Canolty and Knight 2010; Palva and Palva 2017). According to the Binary Hierarchy Brain Body Oscillation Theory (BHBBOT), areas that oscillate at different frequencies can transiently couple by forming harmonic cross-frequency ratios (eg 2:1, 3:1, 4:1, etc.) and decouple by forming nonharmonic irrational ratios (eg 1.6:1) (Klimesch 2013, 2018; Rassi et al. 2019). This prediction is based on mathematical analysis showing that while harmonic ratios would lead to stable phase relationships and therefore enhance “coupling,” those given by irrational numbers (such as the golden mean) would lead to variable phase relationships thereby facilitating a “decoupled state” (Pletzer et al. 2010).

Cross-frequency ratios between alpha and theta rhythms have been investigated in relation to different mental states and cognitive functions (Rodriguez-Larios, Faber, et al. 2020; Rodriguez-Larios, Wong, et al. 2020; Rodriguez-Larios and Alaerts 2019a; Rodriguez-Larios and Alaerts 2020). In the context of working memory, alpha and theta rhythms showed an increased occurrence of cross-frequency ratios around 2:1 and a decreased occurrence of cross-frequency ratios below 1.7:1 during memory retention (Rodriguez-Larios and Alaerts 2019a). This was in line with previous literature showing increases in alpha frequency (Haegens et al. 2014a; Mierau et al. 2017) and decreases in theta frequency (Senoussi et al. 2022) during working memory delays. Although according to the BHBBOT (Klimesch 2018), the harmonic (2:1) frequency configuration of alpha and theta rhythms during working memory would reflect greater coupling between the brain areas generating these rhythms, this prediction has not been empirically tested.

Information theory provides a model-free framework for quantifying (nonlinear) statistical dependencies between different brain regions (Vergara et al. 2017; Fagerholm et al. 2023). Among the most used metrics in neuroscience are mutual information (MI) and transfer entropy (TE) (Timme and Lapish 2018). MI quantifies the shared information between two signals and is typically used to assess functional connectivity, while TE captures the directed, time-lagged influence of one signal on another, making it suitable for assessing effective connectivity (Lindner et al. 2011; Vicente et al. 2011). Both of these metrics are based on the concept of entropy, which measures the level of uncertainty or unpredictability associated with a random variable (Lu and Rodriguez-Larios 2022; Fagerholm et al. 2023). MI uses entropy to quantify the reduction in uncertainty about one signal given knowledge of another, effectively measuring how much information is shared between two signals. TE extends this by quantifying how much the past state of one signal reduces the uncertainty of the future state of another signal, conditioned on the target’s own past—thereby capturing directional, time-lagged interactions.

The Theory of Weakly Coupled Oscillators (TWCO) offers a mechanistic framework for understanding brain connectivity based on transient phase synchrony between oscillators with different natural frequencies (Kuramoto 1991; Ermentrout and Kleinfeld 2001; Breakspear et al. 2010). In this view, synchronization is a nonstationary and nonlinear process that, based on frequency modulations, allows groups of oscillators to coordinate around a preferred phase-relation (Lowet et al. 2022). This theory predicts that synchrony between oscillators depends on the interaction between two parameters: coupling strength and frequency detuning (Hoppensteadt and Izhikevich 1998; Lowet et al. 2022). While coupling strength reflects phase adjustments, detuning is equivalent to mean frequency difference (or numerical ratio) between oscillators. Previous work has shown that gamma band oscillations originating from neural populations in monkey visual cortex behave like weakly coupled oscillators (Lowet et al. 2017). Based on these results, it was proposed that TWCO could be applied to other brain areas and brain rhythms (Lowet et al. 2017, 2022).

In the present study, we aim to determine whether the previously observed shift towards a harmonic (2:1) alpha:theta cross-frequency ratio during working memory retention (Rodriguez-Larios and Alaerts 2019a) is associated with changes in connectivity between the regions generating these rhythms. For this purpose, we re-analyze an existing dataset in which EEG was recorded while participants (*n* = 26) performed a visual working memory task (Rodriguez-Larios and Haegens 2023a). We first estimate the main brain generators of alpha and theta rhythms via spatio-spectral decomposition (Nikulin et al. 2011). Then, we assess whether putative changes in cross-frequency ratios between brain regions generating these rhythms are accompanied by connectivity modulations as assessed through model-free metrics from information theory (MI and TE) and model-based parameters derived from TWCO (detuning and coupling strength). These analyses allow us to directly test whether increased inter-areal connectivity can be inferred from higher “harmonicity” (eg increased occurrence of 2:1 alpha:theta cross-frequency ratios) (Rodriguez-Larios, Wong, et al. 2020; Rodriguez-Larios and Alaerts 2020) as predicted by the BHBBOT (Klimesch 2018).

## Methods

This study used a publicly available EEG dataset that was originally published by Rodriguez-Larios and Haegens (2023). The dataset can be found at https://osf.io/fkhnb/ and MATLAB code of the entire analysis pipeline can be found here: https://osf.io/d9rc7/?view_only=26941f4be9744c5691863bef92b7ea9c.

### Participants

The dataset comprises data from 31 healthy adult volunteers (13 male), with a mean age of 32.5 years (SD = 8.5). All participants had normal or corrected-to-normal vision and no self-reported history of neurological or psychiatric conditions. Participants gave informed consent before taking part in the study. The study protocol and consent procedures were approved by the Institutional Review Board at the New York State Psychiatric Institute (Protocol Number 8001). Participants were compensated $25 per hour for their time. Due to technical issues during EEG acquisition or the presence of unresolvable EEG artifacts, five participants were excluded from analysis, resulting in a final sample size of 26 individuals.

### Design and task

**Fig. 1:**
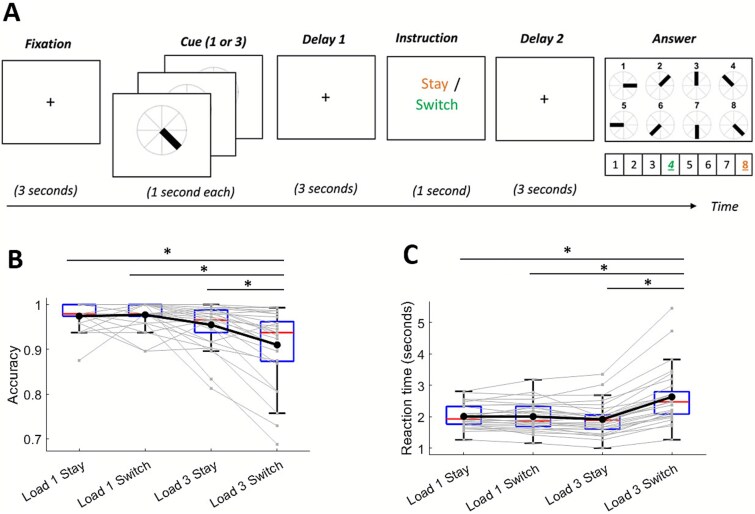
**Working memory task and behavioral results.** A) In each trial, participants were shown either one or three visual stimuli and instructed to memorize their orientations (corresponding to load 1 or load 3, respectively). Following a cue, they were asked to indicate either the original angle (“stay” condition) or its opposite angle (“switch” condition) using the keyboard. In the example shown, colors represent the correct response: orange for “stay” (angle 4) and green for “switch” (angle 4). B and C) Accuracy and reaction time per condition. Asterisks indicate significant differences at *P* < 0.001. Red lines indicate medians and the upper and lower edges of each box indicate the 25th and 75th percentiles, respectively. The whiskers extend to the most extreme data points not considered outliers.

Participants engaged in a visual working memory task (Fig. 1) while EEG was recorded. Each trial began with a 1 s fixation cross. This was followed by the presentation of one or three oriented bars arranged in a circle, with each bar presented sequentially for 1 s each. After a 3-s retention delay, a cue appeared for 1 s, instructing participants to either retain (“stay”) or mentally reverse (“switch”) the presented orientation. Following this instruction, a response-mapping screen appeared, indicating which of the eight numbered buttons corresponded to each possible angle. This mapping changed on every trial. After participants responded, feedback was provided in the form of a green (correct) or red circle (incorrect). The full session lasted approximately one h. For the current study, we only analyzed data from Fixation and Delay periods when the load was 3, giving., a total of 96 trials per participant.

Behavioral results have been reported in detail elsewhere (Rodriguez-Larios and Haegens 2023b). In short, participants showed reduced accuracy and slower reaction times under higher cognitive load when memory manipulation was required and vice versa. In other words, load impaired performance only when memory manipulation was present (switch trials) and memory manipulation impaired performance only under high load (load 3 trials).

### E‌EG acquisition and preprocessing

EEG signals were recorded from 96 scalp electrodes using the BrainVision actiCAP system (Brain Products GmbH, Munich, Germany) at a sampling rate of 500 Hz. Electrodes were placed according to the 10 to 20 system, with Cz used as the reference during acquisition. Signal amplification and digitisation were performed using a Brain Products actiCHamp DC amplifier connected to BrainVision Recorder software (version 2.1). Eye movements were recorded using bipolar electrodes placed vertically (above and below the left eye) and horizontally (at the outer canthi of both eyes). Additionally, electrocardiographic activity (ECG) was recorded using bipolar chest electrodes.

Data preprocessing was carried out in MATLAB R2024b using custom scripts based on EEGLAB functions (Delorme and Makeig 2004). The data were band-pass filtered between 1 and 30 Hz and then re-referenced to the common average. Independent Component Analysis (ICA) was applied, and artefactual components were automatically identified and removed using the ICLabel algorithm (Pion-Tonachini et al. 2019) targeting components associated with muscle, ocular, cardiac, or channel noise. Any component showing an absolute correlation above 0.8 with the VEOG, HEOG, or ECG channels was also excluded. Furthermore, the Artifact Subspace Reconstruction (ASR) method was used to detect and correct transient artifacts, with a cutoff threshold set at 20 standard deviations (Chang et al. 2020).

### Separation of frontal theta and parietal alpha rhythms

Previous literature has shown that, in the context of working memory, mid-frontal regions are robust generators of theta oscillations (Onton et al. 2005), while posterior parietal cortex is one of the main generators of alpha rhythms in similar tasks (Haegens et al. 2014b; Rodriguez-Larios et al. 2022a). Based on these previous studies, we focus our analysis on source-localized mid-frontal theta and posterior parietal alpha rhythms, using the Spatio-Spectral Decomposition (SSD) algorithm (Nikulin et al. 2011) (see MATLAB implementation here: https://github.com/svendaehne/matlab_SPoC/blob/master/SSD/ssd.m). In short, SSD maximizes the signal power at a target frequency while minimizing power at neighboring frequencies, thereby enhancing the signal-to-noise ratio (SNR) of oscillatory components. The algorithm uses a generalized eigenvalue decomposition of covariance matrices derived from band-pass and flanking frequency filters. The SSD output of is a set of spatial filters that optimally project the multichannel EEG data to extract components with maximal power at a specific frequency band and minimal power in neighboring frequency bins.

**Fig. 2:**
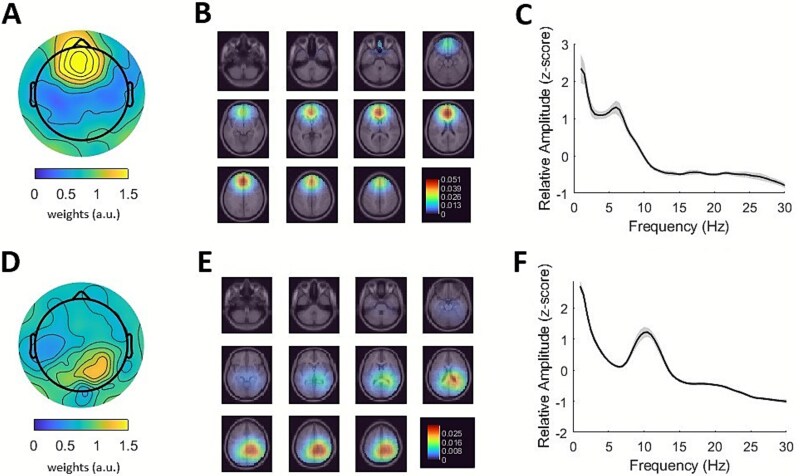
**Frontal theta and parietal alpha components.** A) Mean topography of selected frontal theta components. The absolute values of the spatial patterns given by the SSD algorithm were averaged across subjects. B) Dipole density of selected frontal theta components. Heatmap reflects the normalized density of dipoles in each voxel. C) Average spectrum of selected frontal theta components. Amplitude was normalized in the frequency dimension (z-score) per subject and component. Shaded area depicts standard error. D) Mean topography, E) dipole density and F) average spectrum of selected parietal alpha components. Conventions as in A to C.

In order to differentiate between frontal theta and parietal alpha components, the sources of SSDs were estimated using the DIPFIT plugin implemented in EEGLAB. For the identification of the midfrontal theta component, we selected the dipole with highest SNR in medial prefrontal and anterior cingulate regions (including the rostral and caudal anterior cingulate, superior frontal, medial orbitofrontal, frontal pole, and paracentral areas from the Desikan–Killiany atlas). To identify the parietal alpha component, we selected the parietal dipole (including the superior and inferior parietal lobules, precuneus, and supramarginal gyrus) with the highest SNR. Figure 2 shows the average topography, dipole density and spectrum of frontal theta and parietal alpha components**.**

### Estimation of phase locking and TWCO parameters

**Fig. 3:**
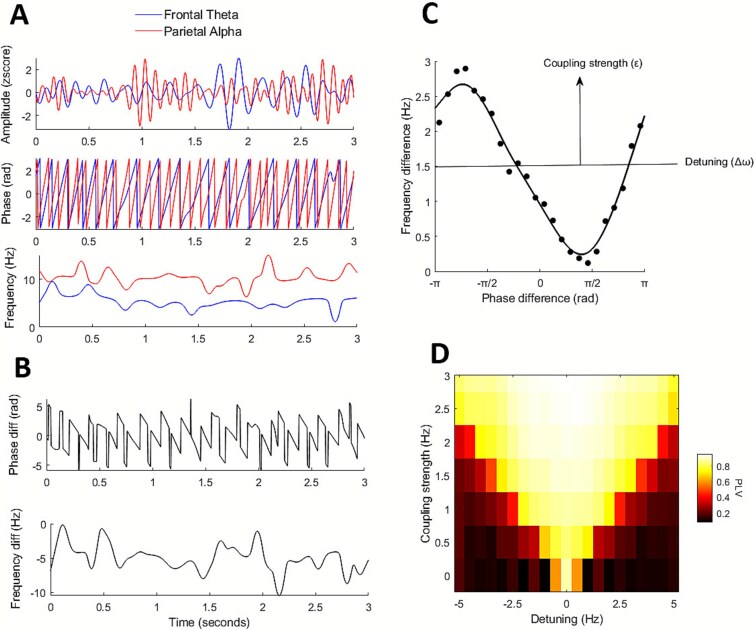
**Schematic depiction of TWCO parameter estimation.** A) Amplitude of alpha and theta components in one trial after bandpass filtering (top), their instantaneous phase (middle) and instantaneous frequency (bottom). B) Differences between the instantaneous phases (top) and frequencies (bottom) of alpha and theta components. C) Schematic depiction of the phase response curve (PRC). To obtain TWCO parameters (coupling strength and detuning), frequency differences are plotted as a function of phase differences across trials and a smoothing spline is fitted to the data. Detuning is taken as the mean level of the fitted function (horizontal line) and coupling strength is taken as its amplitude (vertical arrow). D) Arnold tongue illustrating the relationship between coupling strength, detuning and phase locking value (PLV). Greater coupling strength and lower detuning leads to increased phase locking between oscillators.

To assess connectivity between frontal theta and parietal alpha components, we computed phase locking value (PLV) and the parameters coupling strength and detuning, derived from TWCO. For each component, data were first filtered using zero-phase FIR filters (4 to 8 Hz for frontal theta and 8 to 14 Hz for parietal alpha; Fig. 3A). Then instantaneous phase and frequency were computed through the Hilbert transform, with instantaneous frequency estimated using a Savitzky–Golay derivative filter to smooth phase trajectory and avoid outliers (Schafer 2011; Lowet et al. 2017) (Fig. 3B). PLV was quantified as the mean vector length of phase differences (averaged over a sliding-window of 300-ms with 50-ms steps) within each trial. Coupling strength and detuning were estimated from the TWCO phase response curves (PRCs; Fig. 3C), constructed by binning phase differences (24 π/12 bins) and calculating median frequency differences per bin, with all data pooled across trials (Lowet et al. 2017). PRCs were smoothed with a circular moving average, and a smoothing spline was fitted to the data. Detuning was defined as the mean frequency difference across the PRC, while coupling strength was estimated as the peak-to-trough range of the fitted PRC, reflecting the amplitude of the phase–frequency interaction. All analyses were performed in MATLAB using custom scripts.

### Estimation of cross-frequency ratios

**Fig. 4:**
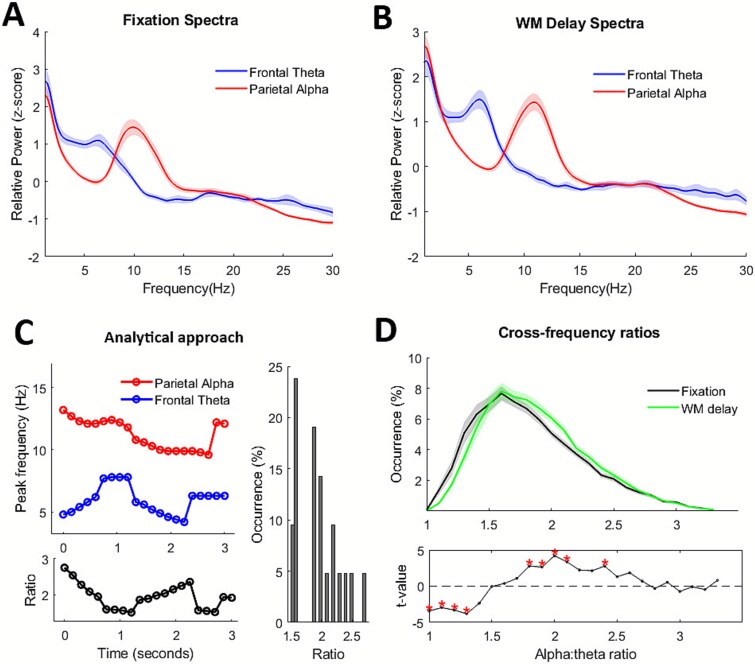
**Alpha:theta cross-frequency ratios during working memory.** A and B) Power spectra of frontal theta (blue) and parietal alpha (red) components during A) fixation and B) working memory (WM) delay. C) Depiction of analytical approach. For each trial and timepoint, the numerical ratio between alpha and theta components’ peak frequencies are estimated. Occurrence (%) can then be compared between conditions. D) Relative occurrence of different alpha:theta cross-frequency ratios in each condition (top) and t-values resulting from the comparison (bottom). Significant ratios (*P* < 0.05 after FDR correction) are marked with red asterisks.

Modulations in alpha:theta cross-frequency ratios were estimated as in Rodriguez-Larios and Alaerts (2019). First, data was transformed with short term Fourier transform (1-s window, 90% overlap, 0.1-Hz resolution between 1 and 30 Hz) using the MATLAB function *spectrogram*. Then, transient peaks for the theta and alpha components were detected using the MATLAB function *findpeaks*. Only peaks with amplitude above an estimate of the 1/f trend were used. This estimate of the 1/f trend was computed by linear fitting in log–log space of the spectrum per component across conditions (Rodriguez-Larios and Haegens 2023a). The proportion of different cross-frequency ratios (from 1 to 3.5) were estimated by dividing transient alpha and theta peak frequencies and rounding to the first decimal (Fig. 4C).

### Information theory metrics

MI and TE between frontal theta and parietal alpha components were estimated following the procedures described by Timme and Lapish (2018). To compute MI, time series of the theta and alpha components were first discretised using a uniform count binning procedure to maximize sensitivity to interactions between the signals. MI was then computed as the reduction in uncertainty of one component given the state of the other, using the standard definition based on joint and marginal probability distributions. TE was estimated in both directions (frontal theta → parietal alpha; parietal alpha → frontal theta) using the conditional MI formulation of TE. A range of neurophysiologically plausible lags was explored by computing TE for delays corresponding to 1 to 20 samples (4 to 80 ms at a sampling rate of 250 Hz), as recommended to account for unknown interaction delays (Timme and Lapish 2018). TE values were then averaged across these lags to obtain a robust measure of directed information flow. All information-theoretic analyses were implemented using the MATLAB code provided in the Neuroscience Information Theory Toolbox (https://github.com/nmtimme/Neuroscience-Information-Theory-Toolbox).

### Statistical analysis

Condition-related differences for each parameter were assessed using paired-samples *t*-tests (as implemented in MATLAB), with corresponding effect sizes calculated using Cohen’s *d* (Nakagawa and Cuthill 2009). To examine the relationship between EEG-derived parameters and behavioral performance (accuracy and reaction time), a multiple linear regression analysis was conducted. Specifically, regression analyses were implemented in MATLAB using the *stepwiselm* function. This approach performs a stepwise linear regression by iteratively adding or removing predictors based on their contribution to model fit, evaluated using the Bayesian Information Criterion (BIC). Significance level for all tests was established at *P* < 0.05. Multiple comparison correction was performed through False Discovery Rate (Benjamini and Hochberg 1995).

## Results

### Alpha:theta cross-frequency ratios

Parietal alpha components showed a significant increase in frequency (*t*(25) = 3.08, *P* = 0.005, *d* = 0.60) and a significant decrease in amplitude (*t*(25) = −2.49, *P* = 0.020, *d* = 0.49) during the working memory delay relative to fixation. In contrast, frontal theta components showed a significant decrease in frequency (*t*(25) = −2.38, *P* = 0.026, *d* = 0.47) and a significant increase in amplitude (*t*(25) = 3.55, *P* = 0.002, *d* = 0.70) during the working memory delay compared to fixation (Fig. 4A and B). In terms of cross-frequency ratios, the working memory delay showed a significant increase in ratios between 1.8 and 2.1 and at 2.4, and a decrease in ratios between 1.0 and 1.3 (*P* < 0.05, FDR-corrected; Fig. 4D) relative to fixation.

To assess whether alpha-theta cross-frequency ratios also changed as a function of cognitive load during the WM delay, we compared the two conditions with largest differences in accuracy and reaction time (ie load 3 switch trials vs. load 1 stay trials). No significant differences were found (all FDR-corrected p-values > 0.05).

### Information theory metrics

**Fig. 5:**
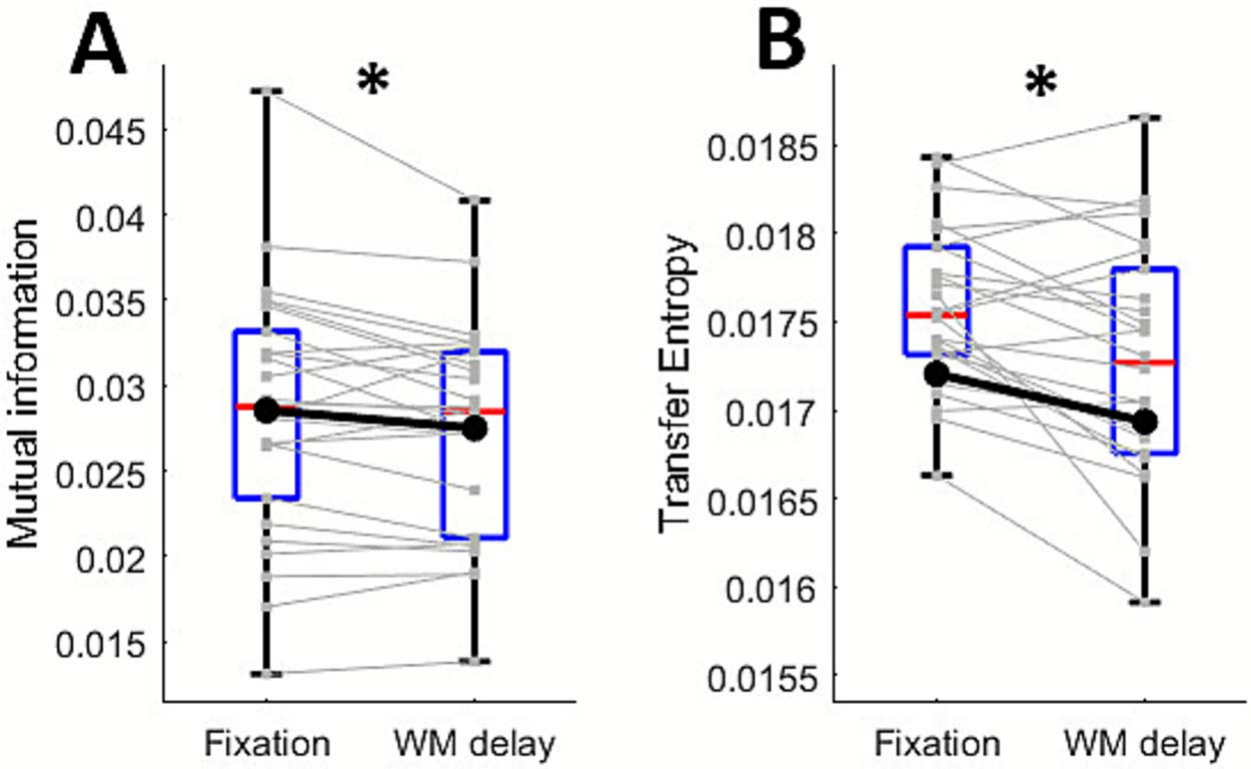
**Condition-related modulations in information theory metrics.** A) Mutual information and B) transfer entropy in the anterior–posterior direction were significantly reduced during working memory delay (relative to fixation). Individual lines reflect subjects, the blue boxes indicate the 25th and 75th percentiles and red centrelines show the median in each condition. Asterisks indicate statistical significance at *P* < 0.05.

Mutual information was significantly reduced during the working memory delay compared to fixation (*t*(25) = −2.29, *P* = 0.031, *d* = 0.45; Fig. 5A). Similarly, TE in the anterior-to-posterior direction was significantly reduced (*t*(25) = −3.34, *P* = 0.003, *d* = 0.65; Fig. 5B), while no significant differences were found in the posterior-to-anterior direction (*t*(25) = −0.90, *P* = 0.377, *d* = 0.18).

### Phase locking and TWCO parameters

**Fig. 6:**
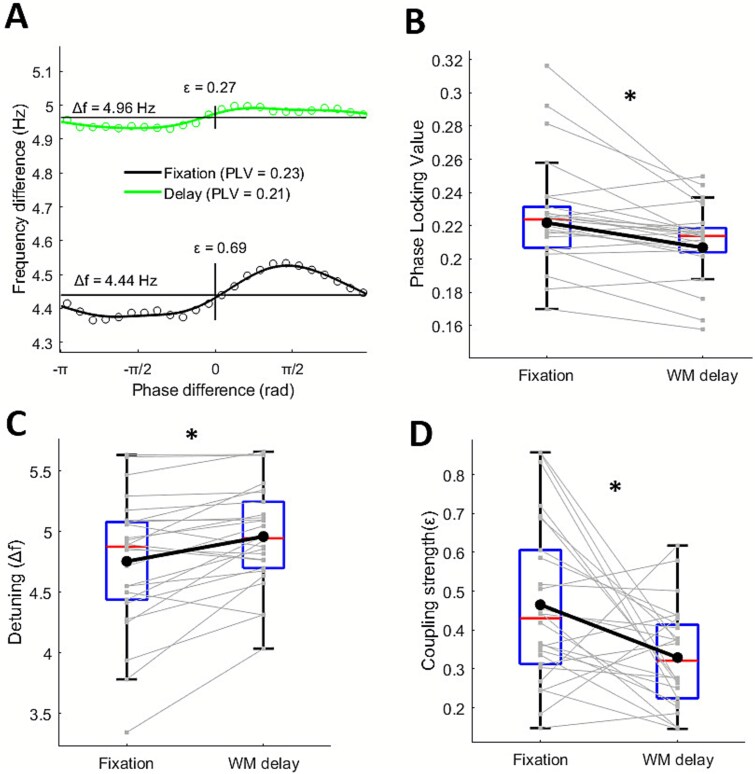
**Modulations in phase locking and TWCO parameters.** A) Phase response curve of a representative subject for fixation and working memory delay. Instantaneous frequency differences between frontal theta and parietal alpha rhythms are plotted as a function of their phase differences. Detuning (Δf) was defined as the mean frequency difference across the PRC, while coupling strength was estimated as the peak-to-trough range of the fitted PRC (ε). B) Boxplots showing condition-related modulations in PLV, C) detuning and D) coupling strength. Individual lines reflect subjects and asterisks indicate significant differences at *P* < 0.05.

To test interactions between frontal theta and parietal alpha rhythms based on TWCO, we first estimated PRCs for each participant and condition (see a representative participant in Fig. 6A). Next, we quantified two key parameters of oscillator coupling: coupling strength and detuning (ie intrinsic frequency difference). Coupling strength was significantly reduced during the working memory delay relative to fixation (*t*(25) = −2.86, *P* = 0.008*, d* = 0.56), and detuning was significantly increased (*t*(25) = 3.76, *P* < 0.001, *d* = 0.73) (Fig. 6C and D). Together these changes make synchronization less likely during the working memory delay. Accordingly, PLV was significantly lower during the working memory delay compared to fixation (*t*(25) = −3.53, *P* = 0.001, *d* = 0.69) (Fig. 6B).

### Relation to performance

**Fig. 7:**
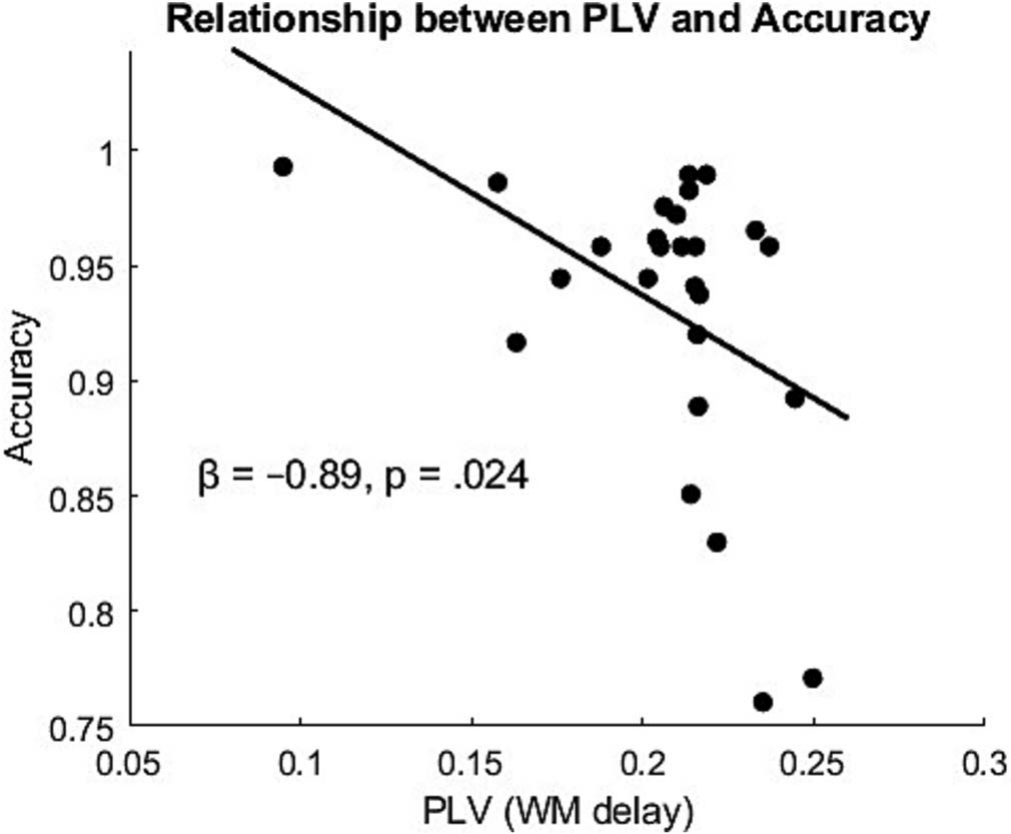
**Relationship between PLV during the working memory delay and behavioral accuracy.** Each point represents one participant. A significant negative linear association was observed (β = −0.89, *P* = 0.024), indicating that stronger phase locking between frontal theta and parietal alpha components was associated with lower task accuracy. Solid line shows the least-squares linear fit.

To identify which neural metrics that best predicted behavioral performance, we conducted two stepwise linear regression analyses with accuracy and reaction time as the dependent variables and nine candidate predictors: PLV, detuning, coupling strength, alpha:theta mean ratio, parietal alpha frequency, frontal theta frequency, TE (anterior to posterior and posterior to anterior), and MI. While no significant predictors were identified for reaction time, PLV was a significant predictor of accuracy (*t*(25) = −2.40, *P* = 0.024). The regression coefficient for PLV was negative (β = −0.89, *SE* = 0.037; Fig. 7), indicating that higher PLV values during the working memory delay were associated with lower accuracy. None of the other predictors significantly contributed to the model.

## Discussion

This study aimed to assess whether the previously reported increase in alpha:theta harmonicity (ie greater occurrence of transient 2:1 frequency ratios) during working memory retention (Rodriguez-Larios and Alaerts 2019b) is accompanied by changes in inter-areal connectivity. This analysis was motivated by a recent theory predicting that a relative increase in harmonicity between brain rhythms oscillating at different frequencies (eg 12 Hz alpha/6 Hz theta = 2) is reflective of a relative increase in connectivity between their brain generators. To assess this hypothesis, we estimated both cross-frequency dynamics and different connectivity metrics between brain sources generating EEG alpha and theta rhythms in the context of a visual working memory task. For connectivity metrics, we included both model-free metrics from IF and model-based parameters derived from the TWCO. In line with previous literature, our results showed that working memory retention (relative to pre-stimulus fixation) was associated with an increased occurrence of alpha:theta cross-frequency ratios around 2:1 and a decrease of ratios closer to 1:1. These cross-frequency modulations were due to a general increase in alpha frequency and decrease in theta frequency. Crucially, the reported (cross)-frequency dynamics were accompanied by a decrease in inter-areal connectivity as quantified by Information theory metrics (ie reduced MI and TE) and TWCO parameters (reduced coupling strength and increased detuning). Finally, PLV (a standard measure of synchrony) was significantly reduced during working memory delay and negatively correlated with behavioral performance. Together, our results suggest that the separation of frontal theta and parietal alpha rhythms in the frequency domain to form a ~ 2:1 cross-frequency ratio during working memory retention reflects functional segregation rather than functional connectivity between their respective neural generators.

According to the Binary Hierarchy Brain Body Oscillations Theory (BHBBOT), a shift towards harmonic cross-frequency configuration reflects a relative connectivity increase between their brain sources (Klimesch 2018). This idea is based on mathematical analysis showing that harmonic ratios between brain rhythms (eg 2:1, 3:1, 4:1) may lead to more stable excitatory phase meetings than nonharmonic ratios (such as the golden mean; ie 1.618… etc)(Pletzer et al. 2010). Contrary to this prediction, we here show that the shift towards 2:1 alpha:theta ratios during working memory is accompanied by decreases (rather than increases) in connectivity between their brain sources, as quantified through information theory metrics. Our interpretation is that rhythms with different peak frequencies that are sufficiently close in the frequency domain can transiently couple by reducing their frequency difference. In this view, a greater occurrence of lower cross-frequency ratios (<2:1) will reflect relative increases in connectivity. Based on our results and previous literature, we hypothesize that alpha-theta cross-frequency dynamics can be better understood as weakly coupled oscillators (Lowet et al. 2017, 2022).

The TWCO conceptualizes synchronization as a nonstationary and nonlinear process in which modulations in frequency difference between pairs of oscillators allow coordination around a preferred phase-relation. Within this framework, phase synchrony is not maximized through fixed harmonic relationships (as in the BHBBOT; Klimesch 2018), but through transient reductions in frequency differences (ie detuning) that enable short-lived but functionally relevant periods of phase coupling. Our findings align with this view, showing rapid changes in frequency difference as a function of phase difference, characterized as the PRC (see Fig. 6A). Importantly, both parameters extracted from the PRC suggested relative decreases in connectivity during working memory retention (increased detuning and reduced coupling strength). This idea was further supported by the significant reductions in phase synchrony, mutual information and TE. Together, our results suggest that alpha and theta rhythms follow dynamic coupling mechanisms based on frequency matching (as predicted by the TWCO), rather than relying on fixed harmonic relationships (as predicted by the BHBBOT).

Given our research question, we limited the connectivity analysis to the main cortical generators of frontal theta and parietal alpha rhythms using data-driven spatial filters (Nikulin et al. 2011). In this regard, it is important to note that different networks (eg Default Mode Network, Salience Network, Dorsal Attention Network) have been shown to contribute to the generation of frontal theta and parietal alpha (Mantini et al. 2007; Ros et al. 2013; Marino et al. 2019; Zuure et al. 2020; Beldzik et al. 2022) and that this might have contributed to previous inconsistencies regarding EEG-derived fronto-parietal connectivity during working memory (Dimitriadis et al. 2016; Dai et al. 2017; Duma et al. 2019; Harding et al. 2015; Riddle et al. 2024a; R. Wang et al. 2019). That is, it is possible that frontal theta and parietal alpha rhythms are generated by different networks (with different natural frequencies) during fixation vs. working memory delay (Barzegaran et al. 2017; Sokoliuk et al. 2019; Rodriguez-Larios et al. 2022b). In support of this idea, we show that the TWCO-derived parameter coupling strength, which has been previously linked to anatomical connectivity (Lowet et al. 2017), was significantly reduced from pre-stimulus fixation to working memory delay, thereby suggesting the involvement of a different network. Nonetheless, it is also possible that coupling strength is modulated by other factors such as shared input or synaptic efficiency (Penn et al. 2016; Engel et al. 2021). Therefore, future studies combining EEG with other methods with greater spatial resolution (Iannaccone et al. 2015; Fahimi Hnazaee et al. 2020; Leicht et al. 2025) are needed to assess whether the generators of frontal theta and parietal alpha rhythms are different during fixation vs. working memory delay.

Although our results show that the specific regions generating frontal theta and parietal alpha rhythms become “decoupled” during working memory retention, this does not necessarily mean that fronto-parietal connectivity is generally reduced. In fact, increases in fronto-parietal connectivity during working memory have been reported using different neuroimaging methods (D’Esposito and Postle 2015). It is possible that other frontal and parietal regions become more connected during working memory by synchronizing at different frequencies. For example, increases in connectivity as a function of working memory load have been reported within the theta and beta bands in both human and nonhuman primates (Salazar et al. 2012; Antzoulatos and Miller 2016; Riddle et al. 2024b). Future studies are needed to assess whether oscillations with other peak frequencies and originating from other areas also behave like weakly coupled oscillators during different cognitive processes.

In summary, we show that the previously reported increase in harmonicity between frontal theta and parietal alpha rhythms during working memory retention is accompanied by decreases in connectivity between the regions generating these rhythms. Hence, our results demonstrate that, contrary to the BHBBOT predictions, harmonic cross-frequency ratios in EEG can reflect decreases (rather than increases) in connectivity.

## References

[ref1] Antzoulatos EG, Miller EK. 2016. Synchronous beta rhythms of frontoparietal networks support only behaviorally relevant representations. elife. 5:e17822. 10.7554/eLife.17822. 27841747 PMC5148609

[ref2] Barzegaran E, Vildavski VY, Knyazeva MG. 2017. Fine structure of posterior alpha rhythm in human EEG: frequency components, their cortical sources, and temporal behavior. Sci Rep. 7:8249. 10.1038/s41598-017-08421-z. 28811538 PMC5557761

[ref3] Beldzik E, Ullsperger M, Domagalik A, Marek T. 2022. Conflict- and error-related theta activities are coupled to BOLD signals in different brain regions. NeuroImage. 256:119264. 10.1016/j.neuroimage.2022.119264. 35508215

[ref4] Benjamini Y, Hochberg Y. 1995. Controlling the false discovery rate: a practical and powerful approach to multiple testing. J R Stat Soc Ser B Methodol. 57:289–300. 10.1111/j.2517-6161.1995.tb02031.x.

[ref5] Breakspear M, Heitmann S, Daffertshofer A. 2010. Generative models of cortical oscillations: neurobiological implications of the Kuramoto model. Front Hum Neurosci. 4:190. 10.3389/fnhum.2010.00190. PMC2995481 21151358

[ref6] Buzsáki G, Draguhn A. 2004. Neuronal oscillations in cortical networks. Science. 304:1926–1929. 10.1126/science.1099745. 15218136

[ref7] Canolty RT, Knight RT. 2010. The functional role of cross-frequency coupling. Trends Cogn Sci. 14:506–515. 10.1016/j.tics.2010.09.001. 20932795 PMC3359652

[ref8] Capilla A et al. 2021. The natural frequencies of the resting human brain: an MEG-based atlas. NeuroImage. 258:119373. 10.1016/j.neuroimage.2022.119373. 35700947

[ref9] Chang C-Y, Hsu S-H, Pion-Tonachini L, Jung T-P. 2020. Evaluation of artifact subspace reconstruction for automatic artifact components removal in Multi-Channel EEG recordings. IEEE Trans Biomed Eng. 67:1114–1121. 10.1109/TBME.2019.2930186. 31329105

[ref10] Christophel TB, Klink C, Spitzer B, Roelfsema PR, Haynes JD. 2017. The distributed nature of working memory. Trends Cogn Sci. 21:111–124. 10.1016/j.tics.2016.12.007. 28063661

[ref11] D’Esposito M, Postle BR. 2015. The cognitive neuroscience of working memory. Annu Rev Psychol. 66:115–142. 10.1146/annurev-psych-010814-015031. 25251486 PMC4374359

[ref12] Dai Z et al. 2017. EEG cortical connectivity analysis of working memory reveals topological reorganization in theta and alpha bands. Front Hum Neurosci. 11:237. 10.3389/fnhum.2017.00237. PMC5427143 28553215

[ref13] Delorme A, Makeig S. 2004. EEGLAB: an open sorce toolbox for analysis of single-trail EEG dynamics including independent component anlaysis. J Neurosci Methods. 134:9–21. 10.1016/j.jneumeth.2003.10.009. 15102499

[ref14] Dimitriadis S, Sun Y, Thakor NV, Bezerianos A. 2016. Causal interactions between frontalθ – parieto-occipitalα2 predict performance on a mental arithmetic task. Front Hum Neurosci. 10:454. 10.3389/fnhum.2016.00454. 27683547 PMC5022172

[ref15] Duma GM et al. 2019. Functional dissociation of anterior cingulate cortex and intraparietal sulcus in visual working memory. Cortex. 121:277–291. 10.1016/j.cortex.2019.09.009. 31669977

[ref16] Engel TA, Schölvinck ML, Lewis CM. 2021. The diversity and specificity of functional connectivity across spatial and temporal scales. NeuroImage. 245:118692. 10.1016/j.neuroimage.2021.118692. 34751153 PMC9531047

[ref17] Ermentrout GB, Kleinfeld D. 2001. Traveling electrical waves in cortex: insights from phase dynamics and speculation on a computational role. Neuron. 29:33–44. 10.1016/S0896-6273(01)00178-7. 11182079

[ref18] Fagerholm ED, Dezhina Z, Moran RJ, Turkheimer FE, Leech R. 2023. A primer on entropy in neuroscience. Neurosci Biobehav Rev. 146:105070. 10.1016/j.neubiorev.2023.105070. 36736445

[ref19] Fahimi Hnazaee M et al. 2020. Localization of deep brain activity with scalp and subdural EEG. NeuroImage. 223:117344. 10.1016/j.neuroimage.2020.117344. 32898677

[ref20] Fries P . 2015. Rhythms for cognition: communication through coherence. Neuron. 88:220–235. 10.1016/j.neuron.2015.09.034. 26447583 PMC4605134

[ref21] Haegens S, Cousijn H, Wallis G, Harrison PJ, Nobre AC. 2014. Inter- and intra-individual variability in alpha peak frequency. 92:46–55. 10.1016/j.neuroimage.2014.01.049. PMC4013551 24508648

[ref22] Harding IH, Yücel M, Harrison BJ, Pantelis C, Breakspear M. 2015. Effective connectivity within the frontoparietal control network differentiates cognitive control and working memory. NeuroImage. 106:144–153. 10.1016/J.NEUROIMAGE.2014.11.039. 25463464

[ref23] Hoppensteadt FC, Izhikevich EM. 1998. Thalamo-cortical interactions modeled by weakly connected oscillators: could the brain use FM radio principles? Biosystems. 48:85–94. 10.1016/S0303-2647(98)00053-7. 9886635

[ref24] Iannaccone R et al. 2015. Conflict monitoring and error processing: new insights from simultaneous EEG-fMRI. NeuroImage. 105:395–407. 10.1016/j.neuroimage.2014.10.028. 25462691

[ref25] Klimesch W . 2013. An algorithm for the EEG frequency architecture of consciousness and brain body coupling. Front Hum Neurosci. 7:766. 10.3389/fnhum.2013.00766. 24273507 PMC3824085

[ref26] Klimesch W . 2018. The frequency architecture of brain and brain body oscillations: an analysis. Eur J Neurosci. 48:2431–2453. 10.1111/ejn.14192. 30281858 PMC6668003

[ref27] Kuramoto Y . 1991. Collective synchronization of pulse-coupled oscillators and excitable units. Physica D: Nonlinear Phenomena. 50:15–30. 10.1016/0167-2789(91)90075-K.

[ref28] Lea-Carnall CA, Montemurro MA, Trujillo-Barreto NJ, Parkes LM, El-Deredy W. 2016. Cortical resonance frequencies emerge from network size and connectivity. PLoS Comput Biol. 12:e1004740. 10.1371/journal.pcbi.1004740. 26914905 PMC4767278

[ref29] Leicht G et al. 2025. Simultaneous EEG-fMRI reveals a visual working memory encoding network related to theta oscillatory activity in healthy subjects. Hum Brain Mapp. 46:e70216. 10.1002/hbm.70216. 40256822 PMC12010137

[ref30] Lindner M, Vicente R, Priesemann V, Wibral M. 2011. TRENTOOL: a Matlab open source toolbox to analyse information flow in time series data with transfer entropy. BMC Neurosci. 12:1–22. 10.1186/1471-2202-12-119/FIGURES/9. 22098775 PMC3287134

[ref31] Lowet E, Roberts MJ, Peter A, Gips B, De Weerd P. 2017. A quantitative theory of gamma synchronization in macaque V1. elife. 6:1–44. 10.7554/eLife.26642.001. PMC5779232 28857743

[ref32] Lowet E, De Weerd P, Roberts MJ, Hadjipapas A. 2022. Tuning neural synchronization: the role of variable oscillation frequencies in neural circuits. Front Syst Neurosci. 16:908665. 10.3389/FNSYS.2022.908665. PMC9304548 35873098

[ref33] Lu Y, Rodriguez-Larios J. 2022. Nonlinear EEG signatures of mind wandering during breath focus meditation. Current Research in Neurobiology. 3:100056. 10.1016/J.CRNEUR.2022.100056. 36518347 PMC9743068

[ref34] Mantini D, Perrucci MG, Del Gratta C, Romani GL, Corbetta M. 2007. Electrophysiological signatures of resting state networks in the human brain. Proc Natl Acad Sci USA. 104:13170–13175. 10.1073/pnas.0700668104. 17670949 PMC1941820

[ref35] Marino M, Arcara G, Porcaro C, Mantini D. 2019. Hemodynamic correlates of electrophysiological activity in the default mode network. Front Neurosci. 13:1060. 10.3389/fnins.2019.01060. 31636535 PMC6788217

[ref36] Mierau A, Klimesch W, Lefebvre M. 2017. Review state-dependent alpha peak frequency shifts: experimental evidence, potential mechanisms and functional implications. Neuroscience. 360:146–154. 10.1016/j.neuroscience.2017.07.037. 28739525

[ref37] Nakagawa S, Cuthill IC. 2009. Effect size, confidence interval and statistical significance: a practical guide for biologists. Biol Rev. 84:515. 10.1111/j.1469-185X.2009.00083.x. 17944619

[ref38] Nikulin VV, Nolte G, Curio G. 2011. A novel method for reliable and fast extraction of neuronal EEG/MEG oscillations on the basis of spatio-spectral decomposition. NeuroImage. 55:1528–1535. 10.1016/j.neuroimage.2011.01.057. 21276858

[ref39] Onton J, Delorme A, Makeig S. 2005. Frontal midline EEG dynamics during working memory. NeuroImage. 27:341–356. 10.1016/j.neuroimage.2005.04.014. 15927487

[ref40] Palva JM, Palva S. 2017. Functional integration across oscillation frequencies by cross-frequency phase synchronization. Eur J Neurosci. 48:2399–2406. 10.1111/ejn.13767. 29094462

[ref41] Penn Y, Segal M, Moses E. 2016. Network synchronization in hippocampal neurons. Proc Natl Acad Sci. 113:3341–3346. 10.1073/pnas.1515105113. 26961000 PMC4812773

[ref42] Pion-Tonachini L, Kreutz-Delgado K, Makeig S. 2019. ICLabel: an automated electroencephalographic independent component classifier, dataset, and website. NeuroImage. 198:181–197. 10.1016/j.neuroimage.2019.05.026. 31103785 PMC6592775

[ref43] Pletzer B, Kerschbaum H, Klimesch W. 2010. When frequencies never synchronize: the golden mean and the resting EEG. Brain Res. 1335:91–102. 10.1016/j.brainres.2010.03.074. 20350536

[ref44] Rassi E, Dorffner G, Gruber W, Schabus M, Klimesch W. 2019. Coupling and decoupling between brain and body oscillations. Neurosci Lett. 711:134401. 10.1016/j.neulet.2019.134401. 31349018

[ref45] Riddle J et al. 2024. Internal representations are prioritized by frontoparietal theta connectivity and suppressed by alpha oscillation dynamics: evidence from concurrent transcranial magnetic stimulation EEG and invasive EEG. J Neurosci. 44:e1381232024. 10.1523/JNEUROSCI.1381-23.2024. 38395616 PMC11007311

[ref46] Rodriguez-Larios J, Alaerts K. 2019. Tracking transient changes in the neural frequency architecture: harmonic relationships between theta and alpha peaks facilitate cognitive performance. J Neurosci. 39:6291–6298. 10.1523/JNEUROSCI.2919-18.2019. 31175211 PMC6687903

[ref47] Rodriguez-Larios J, Alaerts K. 2020. EEG alpha-theta dynamics during mind wandering in the context of breath focus meditation: an experience sampling approach with novice meditation practitioners. Eur J Neurosci. 53:1855–1868. 10.1111/ejn.15073. 33289167

[ref48] Rodriguez-Larios J, Haegens S. 2023. Genuine beta bursts in human working memory: controlling for the influence of lower-frequency rhythms. Advancesin/Psychology. 1:1. 10.56296/aip00006.

[ref49] Rodriguez-Larios J, Faber P, Achermann P, Tei S, Alaerts K. 2020a. From thoughtless awareness to effortful cognition: alpha—theta cross-frequency dynamics in experienced meditators during meditation, rest and arithmetic. Sci Rep. 10:5419. 10.1038/s41598-020-62392-2. PMC7096392 32214173

[ref50] Rodriguez-Larios J, Wong KF, Lim J, Alaerts K. 2020b. Mindfulness training is associated with changes in alpha-theta cross-frequency dynamics during meditation. Mindfulness. 11:2695–2704. 10.1007/s12671-020-01487-3.

[ref51] Rodriguez-Larios J, ElShafei A, Wiehe M, Haegens S. 2022. Visual working memory recruits two functionally distinct alpha rhythms in posterior cortex. eNeuro. 9:ENEURO.0159–ENEU22.2022. 10.1523/ENEURO.0159-22.2022. 36171059 PMC9536853

[ref52] Ros T et al. 2013. Mind over chatter: plastic up-regulation of the fMRI salience network directly after EEG neurofeedback. NeuroImage. 65:324–335. 10.1016/j.neuroimage.2012.09.046. 23022326 PMC5051955

[ref53] Salazar RF, Dotson NM, Bressler SL, Gray CM. 2012. Content-specific Fronto-parietal synchronization during visual working memory. Science. 338:1097–1100. 10.1126/science.1224000. 23118014 PMC4038369

[ref54] Schafer RW . 2011. What is a Savitzky-Golay filter? [lecture notes]. IEEE Signal Process Mag. 28:111–117. 10.1109/MSP.2011.941097.

[ref55] Senoussi M et al. 2022. Theta oscillations shift towards optimal frequency for cognitive control. Nature Human Behaviour 2022 6:7. 6:1000–1013. 10.1038/s41562-022-01335-5. 35449299

[ref56] Sokoliuk R et al. 2019. Two spatially distinct posterior alpha sources Fulfill different functional roles in attention. J Neurosci. 39:7183–7194. 10.1523/JNEUROSCI.1993-18.2019. 31341028 PMC6733553

[ref57] Spyropoulos G, Bosman CA, Fries P. 2018. A theta rhythm in macaque visual cortex and its attentional modulation. Proc Natl Acad Sci USA. 115:E5614–E5623. 10.1073/pnas.1719433115. 29848632 PMC6004461

[ref58] von Stein A, Sarnthein J. 2000. Different frequencies for different scales of cortical integration: from local gamma to long range alpha/theta synchronization. Int J Psychophysiol. 38:301–313. 10.1016/S0167-8760(00)00172-0. 11102669

[ref59] Timme NM, Lapish C. 2018. A tutorial for information theory in neuroscience. eneuro. 5. 10.1523/ENEURO.0052-18.2018. PMC6131830 30211307

[ref60] Varela FJ, Lachaux J-P, Rodriguez E, Martinerie J. 2001. The brainweb: phase synchronization and large-scale integration. Nat Rev Neurosci. 2:229–239. 10.1038/35067550. 11283746

[ref61] Vergara VM, Miller R, Calhoun V. 2017. An information theory framework for dynamic functional domain connectivity. J Neurosci Methods. 284:103–111. 10.1016/j.jneumeth.2017.04.009. 28442296 PMC5553686

[ref62] Vicente R, Wibral M, Lindner M, Pipa G. 2011. Transfer entropy—a model-free measure of effective connectivity for the neurosciences. J Comput Neurosci. 30:45–67. 10.1007/s10827-010-0262-3. 20706781 PMC3040354

[ref63] Vissani M et al. 2025. Spike-phase coupling of subthalamic neurons to posterior perisylvian cortex predicts speech sound accuracy. Nat Commun. 16:3357. 10.1038/s41467-025-58781-8. 40204804 PMC11982203

[ref64] Wang X-J . 2010. Neurophysiological and computational principles of cortical rhythms in cognition. Physiol Rev. 90:1195–1268. 10.1152/physrev.00035.2008. 20664082 PMC2923921

[ref65] Wang R et al. 2019. Consistency and dynamical changes of directional information flow in different brain states: A comparison of working memory and resting-state using EEG. Neuroimage. 203:116188. 10.1016/j.neuroimage.2019.116188. 31533066

[ref66] Zuure MB, Hinkley LB, Tiesinga PHE, Nagarajan SS, Cohen MX. 2020. Multiple midfrontal thetas revealed by source separation of simultaneous MEG and EEG. J Neurosci. 40:7702–7713. 10.1523/JNEUROSCI.0321-20.2020. 32900834 PMC7531541

